# Electrocautery vs. Cold Cutting in Modified Radical Mastectomy: A Systematic Review and Meta-Analysis

**DOI:** 10.3390/jcm14186437

**Published:** 2025-09-12

**Authors:** Dennis Cicio, Alin Gheorghe Balta, Teodora Livia Homorozan, Vladimir Ciornei, Octav Marius Russu, Horea Rares Benea, Mihai Pavel

**Affiliations:** 1 Faculty of Medicine, Iuliu Hatieganu University of Medicine and Pharmacy, 400347 Cluj Napoca, Romania; cicio.dennis@elearn.umfcluj.ro (D.C.); balta.alin.gheorghe@elearn.umfcluj.ro (A.G.B.); homorozan.teodora.livia@elearn.umfcluj.ro (T.L.H.); ciornei_vladimir@elearn.umfcluj.ro (V.C.); 2 Faculty of Medicine, Universitatea de Medicină, Farmacie, Științe și Tehnologie „George Emil Palade”, 540139 Targu Mures, Romania; octav.russu@umfst.ro; 3 Faculty of Medicine, University of Oradea, 410073 Oradea, Romania; pavel.mihai1@student.uoradea.ro

**Keywords:** modified radical mastectomy, blood loss, electrocautery, scalpel, operative time, breast cancer, surgical technique, infection

## Abstract

**Background and Objectives:** Modified radical mastectomy (MRM) is a common surgical procedure, with outcomes that are influenced by the instruments used in the operation. This meta-analysis aimed to compare “cold cutting” or “traditional” techniques and monopolar or bipolar electrocautery. **Materials and Methods:** A comprehensive search of five databases was conducted, with only studies of adult patients undergoing MRM in clearly defined groups selected. Data from 12 RCTs and 3 cohort studies summarizing 1372 participants was extracted and then synthesized using random-effects models. Risk of Bias was assessed for each of the included studies using the RoB-2 or ROBINS-I tool. **Results:** Scalpel or scissor use in dissection and flap raising was associated with a significantly lower risk of seroma formation (LogOR = −0.90, 95% CI: −1.26 to −0.54, *p* < 0.01). Conversely, electrocautery demonstrated advantages including reduced operative time (MD = −13.14 min, 95% CI: −19.58 to −6.70, *p* < 0.01) and decreased intraoperative blood loss (MD = −171.60 mL, 95% CI: −259.35 to −84.41, *p* < 0.01). No statistically significant differences were observed in total drain output (MD = −16.45 mL, 95% CI: −170.96 to 138.06, *p* = 0.83) or duration of drainage (MD = 0.41 days, 95% CI: −0.41 to 1.23, *p* = 0.32). Similarly, rates of infection, ecchymosis, and flap necrosis did not differ significantly between techniques. **Conclusions:** Electrocautery should be employed in patients who benefit from a shorter operative time and lower blood loss, while patients in better clinical condition should benefit from cold cutting techniques. Data on patient-reported outcomes and wound cytokine levels were sparse and inconsistent. This meta-analysis was registered in PROSPERO (ID: CRD420251059886).

## 1. Introduction

Breast cancer is the most common cancer diagnosed in women globally. In 2022, there were an estimated 2.3 million new cases diagnosed and 670,000 deaths attributed to breast cancer [1].

Breast surgeries were first described in the Edwin Smith Egyptian papyrus. A total of 48 cases of traumatic injuries and tumors were recorded in this historical document, in which the majority of the breast tumors were treated with simple cautery [2].

Mastectomies are surgical procedures where all breast gland tissue is removed. Mastectomies are classified based on the quantity and type of tissue removed. For example, simple mastectomy consists of removing all the breast tissue, as well as adjacent skin and the nipple–areola complex. From here, multiple variations in the simple mastectomy evolved, beginning with radical mastectomy, which involves the simultaneous removal of the chest wall muscles and lymphadenectomy. Henceforth, more conservative variations evolved, such as modified radical mastectomy, skin-sparing mastectomy, and nipple-sparing mastectomy. Even though more conservative techniques are now more commonly used, modified radical mastectomies (MRMs) still remain an essential adjuvant therapy for certain benign and malignant breast neoplasms [3].

Cold cutting refers to surgical instruments, usually a scalpel or scissors, or other tools used for cutting or incising tissue without using heat. A scalpel is an essential surgical tool used for incisions, dissections, and a variety of other procedures [4]. Although it is extensively used, because of its precision, minimal adjacent tissue injury, and ease of use, it also comes with disadvantages such as considerable blood loss and collateral injuries to the medical staff.

Monopolar and bipolar electrocautery are types of electrosurgery that use electrical current to cut, ablate, dissect, or coagulate tissue. An electrosurgical unit consists of a generator and a handpiece with one or more electrodes [5].

In bipolar electrocautery, both the active and return electrodes are located at the surgical site. The two tips of the forceps serve as both return and active electrode interchangeably, allowing only the tissue between the tips to be part of the circuit. Since one tip acts as the return electrode, a separate patient return electrode is not needed. This type of electrosurgery can operate in any environment, which is a significant advantage when coagulation is needed in a wet field.

In monopolar electrocautery, the active electrode is used in the surgical site, and the return electrode is placed elsewhere on the patient’s body in order to safely remove current from the patient. The current flows from the active electrode to the return electrode, passing through the patient’s body and completing the circuit [6].

Devices that use electricity were invented to more effectively reduce blood loss by maintaining hemostasis [7].

One aspect that surgeons should remember when performing an MRM is thermal spread from the use of electrocautery, which increases the risk of flap necrosis, wound infection, and prolonged lymphatic drainage [8].

During mastectomy, correct assessment of skin flap perfusion can prevent necrosis, which is a major contributor to infection, delayed healing, and postponed adjuvant therapy. Thus, the need arose for clinical and instrumental methods to determine skin flap perfusion after mastectomy. A 2017 review concluded that certain intraoperative techniques, namely handheld Doppler devices, laser Doppler, fluorescein angiography, and indocyanine green techniques, can be used successfully to detect skin areas that are at risk of necrosis [9].

Of the last enumerated methods, laser-assisted indocyanine green dye angiography (ICG) is the most widely used and presents more advantages, particularly in high-risk patients [10].

Seroma formation is the complication that occurs most frequently following mastectomy and axillary dissection. It entails the buildup of clear fluid in a tissue after an operation [11]. Although it is considered an acceptable complication by surgeons, it can cause other complications such as wound infection, lymphedema [12], flap necrosis, prolonged length of hospital stay, and sepsis, as well as delay in initiation of adjuvant therapy.

A technique that started gaining traction is the tumescent technique or hidrodissection, which implies the dissection of breast tissue by injection of crystalloid fluids. It was demonstrated to have a lower overall complication rate and operative time [13]. Further, another technique used for the prevention of seroma formation is ensuring flap adherence to the axilla and chest wall, eliminating the space left by the operation. Such a technique employs a special kind of suture, named a quilting suture [14], but leads to poor cosmetic outcomes.

Various in-wound “additives” have been investigated. Microporous polysaccharide hemispheres (MPHs) have been proven to accelerate the clotting process, also leading to faster wound healing [15]. Another proven method is fibrin sealants, which reduce the rates and volume of seroma formation [16].

The present meta-analysis aims to compare surgical techniques using a cold scalpel or scissors with electrocautery, focusing on their perioperative outcomes, in female oncological patients undergoing modified radical mastectomy.

The current studies found in the literature have contradictory results; while some reports show improved intraoperative outcomes with electrocautery, these benefits are counterbalanced by increased postoperative complications noted in other studies.

Thus emerges the need for a thorough study that evaluates perioperative outcomes of surgical techniques in modified radical mastectomy to decide which one performs better and leads to fewer complications [7,17].

## 2. Materials and Methods

### 2.1. Search Strategy

The PICO framework was applied to develop an effective search strategy and to guide research questions.

- P (Population): Female patients undergoing modified radical mastectomy.
- I (Intervention): Use of electrocautery (thermal cutting) for raising the flap in mastectomy.
- C (Comparison): Use of scalpel (cold cutting) for raising the flap in mastectomy.
- O (Outcome): Operative time, blood loss, postoperative drainage, seroma formation, surgical site infection, wound dehiscence, pain levels, duration of hospital stay, and risk of short-term and long-term complications.

Following the establishment of the search strategy framework, we initiated the search process by searching on 5 databases (Epistemonikos, MEDLINE (PubMed), Embase, Web of Science, and Proquest).

The search was conducted without time or language limitations.

The last update to the search was made January 2025. The protocol for this systematic review and meta-analysis closely followed the guidelines of the Preferred Reporting Items for Systematic Reviews and Meta-Analysis statement (PRISMA) [18].

Reference lists of cited studies were also examined (snowballing technique) to find other studies that met the inclusion criteria.

Similar systematic reviews and meta-analyses were identified from abstract screening and thoroughly combed for relevant studies.

Further, a crossref metadata search was used to find records with no DOI and other stray records not mentioned in the main databases.

The terms used for the search combined “scalpel”, “knife”, “blade”, “scissor”, “electro”, “mastectomy”, and “breast” to find all the titles that met the inclusion criteria.

The search strings for each individual database can be found in the PROSPERO protocol.

### 2.2. Selection Criteria

All study designs of levels I to IV evidence were considered, randomized controlled trials (RCTs), controlled (non-randomized) clinical trials (CCTs), prospective and retrospective comparative cohort studies, case-control studies, and case series, with a focus on clinically adult patients undergoing modified radical mastectomy. Only published studies were selected, conducting a comparison of two techniques: cold cutting, which may include scalpel or scissor dissection, and electrocautery, represented by either monopolar or bipolar electrocautery.

#### 2.2.1. Inclusion Criteria

Studies prospectively met the following criteria:

1. The population analyzed were adult patients (aged 18 or older);
2. Undergoing modified radical mastectomy (pectoralis muscle sparing);
3. Studies must compare electrocautery with cold cutting dissection in defined groups;
4. Studies included must report at least one relevant outcome from the PICO framework;
5. Use of human subjects.

#### 2.2.2. Exclusion Criteria

Studies of the following structure were excluded:

1. Pre-print;
2. Reviews;
3. Case report;
4. Animal studies;
5. In vitro studies;
6. Editorials.

### 2.3. Study Selection and Data Extraction

Screening was performed at every level by three authors (D.C., T.H., and A.B.) in a blinded way to eliminate inter-observer bias and ensure complete and accurate article selection. The title and abstract of all identified studies were initially reviewed, followed by an in-depth analysis of each full text.

**Figure 1:**
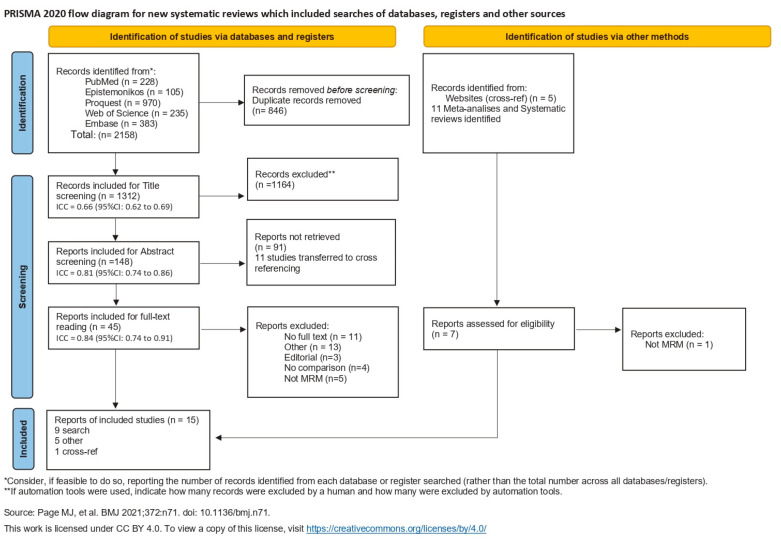
Flowchart of the literature search [19,20,21,22,23,24,25,26,27,28,29,30,31,32,33].

For each level of screening, intra-class correlation (ICC) was calculated. ICC is represented in the Prisma flow chart (Figure 1) at each step of the screening process.

ICC values less than 0.5 are indicative of poor reliability, values between 0.5 and 0.75 indicate moderate reliability, values between 0.75 and 0.9 indicate good reliability, and values greater than 0.90 indicate excellent reliability [34].

Disagreements were settled by a fourth more experienced author (H.B.).

#### Data Extraction Process

Two authors (D.C. and A.B.) independently collected all relevant data present in the studies using a predefined spreadsheet in Microsoft Excel, 2019 version (Microsoft Corporation, Redmond, WA, USA).

Where the data was not clearly reported, in order to not introduce bias through assumption, it was not extracted.

Before the data analysis, the author (H.B.) verified the extracted data of each individual study, ensuring the validity of the results.

### 2.4. Quality Assessment (Risk of Bias and Level of Evidence)

One author (D.C.), under the supervision of a second author (H.B.), conducted an unblinded risk of bias and quality assessment for each included study.

Quality assessment was performed using the scoring protocol developed by the Oxford Centre for Evidence-Based Medicine to determine levels of evidence (LoE). The results are depicted in Table 1.

**Figure 2:**
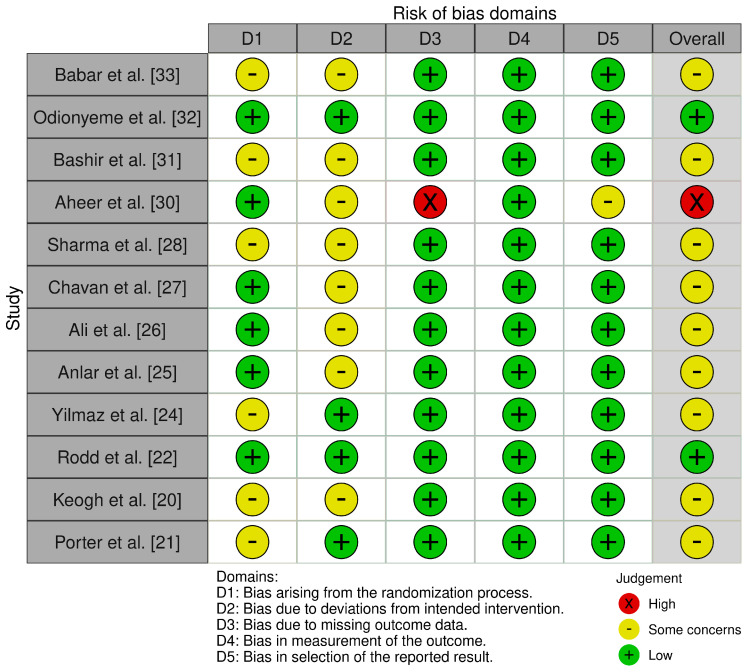
Risk of Bias criteria for randomized surgical studies [20,21,22,24,25,26,27,28,30,31,32,33].

**Figure 3:**
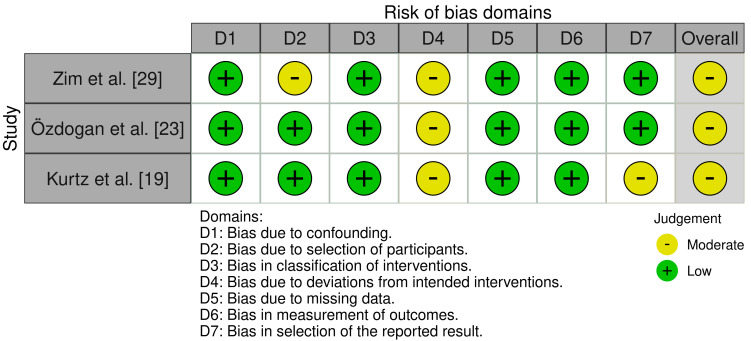
Risk of bias criteria for non-randomized surgical studies [19,23,29].

Risk of Bias assessment was performed using both the RoB 2 tool [35] and the ROBINS-I tool [36] for the included RCTs and cohort studies. Scoring was graded using the in-tool algorithm on a scale from low risk, moderate risk, and high risk on 5 items for the RoB tool, and low risk, moderate risk, severe risk, and critical risk on 7 items for the ROBINS-I tool. The items used to evaluate bias in each trial included bias due to the randomization or participant selection process, deviations from intended interventions or intervention classification, missing outcome data, measurement of outcomes, selection of the reported result, and potential confounding (Figure 2 and Figure 3).

### 2.5. Statistical Analysis and Synthesis Methods

The collected data was represented by the mean, median, standard deviation, and min–max range.

Meta-analyses were conducted using IBM SPSS Statistics (Version 30) and Microsoft Excel. Continuous outcomes were calculated using unstandardized mean difference, with adjustments for unequal group variances, and represented as mean difference (MD) with 95% confidence interval (CI). For binary outcomes, Log odds ratio (logOR) with 95%CI was used to calculate and report effect sizes. All meta-analyses were performed using a random-effects model, with restricted maximum likelihood (REML) selected as the estimator. Two-tailed significance tests were applied for all pooled effect estimates. The graphical representation of statistically relevant results was made using forest plots generated in SPSS.

Representation of statistically insignificant or considered secondary results was performed in tables as pooled, weighted means with corresponding pooled standard errors, as calculated in Excel. Where possible, statistical analysis was carried out.

I2 estimate was computed to estimate heterogeneity between different study outcome groups. The following thresholds for heterogeneity were used [37]: “0–40%, heterogeneity is negligible; 30–60%, moderate heterogeneity; 50–90%, substantial heterogeneity; and 75–100%, considerable heterogeneity”.

Subgroup analysis was performed where high heterogeneity was detected, or when study design suggested an impact on the outcomes reported.

### 2.6. Ethical Approval

Ethical committee approval was not required for this study.

### 2.7. Meta-Analysis Registration

This study was registered on the International Prospective Register of Systematic Reviews [38] (PROSPERO registration number: CRD420251059886).

## 3. Results

### 3.1. Search Strategy and Literature Selection

The first combined search discovered 2158 articles, 846 of which were duplicates and were excluded. Title screening included 1312 articles, of which 1164 were eliminated due to not meeting the study’s inclusion criteria. The remaining 148 articles were included for abstract screening, of which 91 articles were excluded, and 11 articles were determined to be other systematic reviews on the topic and transferred to the cross-ref process. In total, 45 articles entered final full-text exclusion screening. All articles were meticulously analyzed by reading the full text and were excluded for the following reasons: 11 were excluded for lack of full text, 3 for editorial structure, 4 for no comparison between cold cutting and electrocautery, 5 for not analyzing modified radical mastectomy (focusing on other procedures), and 13 for other comparisons.

No additional articles were identified through the reference lists.

Two articles were identified through the cross-ref procedure, and one was excluded after reading the full-text as the procedure examined was not modified radical mastectomy.

Five stray articles were included from the cross-ref metadata search.

After the exclusion process, 15 articles were included in this systematic review (Figure 1).

A significant part of the included articles consisted of RCTs (level IB evidence), while two prospective cohort studies and one was a retrospective cohort study.

### 3.2. Study Characteristics

A total of 1372 patients have been included, of which 730 underwent electrocautery and 642 cold cutting procedures (Table 1).

It is important to highlight that eight studies used electrocautery as a method of coagulation in the scalpel group.

Nine studies opted to extract the drainage tube when the fluid decreased below 30 mL per 24 h; two studies did not document when the drain removal decision was made, while four studies removed the drain after the output dropped below 50 mL per 24 h.

### 3.3. Outcomes

This systematic review synthesized findings from fifteen studies [19,20,21,22,23,24,25,26,27,28,29,30,31,32,33] that compared the impact of using either scalpel or electrocautery in flap raising during the modified radical mastectomy operation. None of the demographics, which included age, BMI, and number of collected or positive lymph nodes, were influenced by the technique used. No statistical analysis could be performed on the stage of disease, follow-up period, or patient risk factors. The results are given in Table 2.

The secondary outcomes included in this meta-analysis were named as such because of the lack of studies reporting them, thus making their results less accurate. They included hospital stay, specimen weight, specimen volume, seroma volume, and flap area. Some of the outcomes did not report standard deviation, making statistical analysis impossible. The results are given in Table 3.

No statistically significant differences were observed between the techniques for the following complications: hematoma, flap necrosis, and ecchymosis. The results are given in Table 4.

The most reported outcomes were as follows: seroma risk, infection risk, blood loss, operative time, total volume drained, and duration of drain.

#### 3.3.1. Seroma Risk

**Figure 4:**
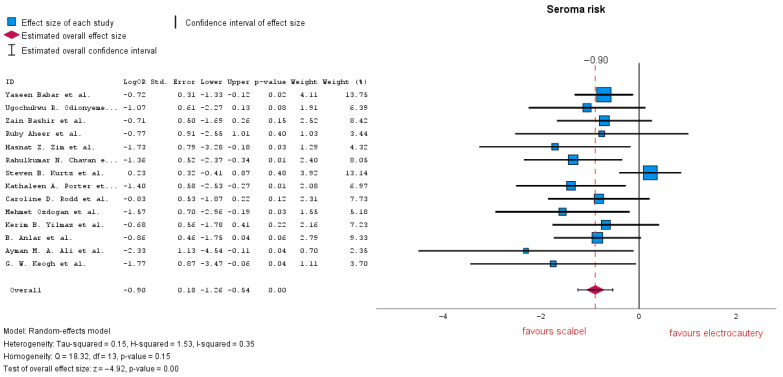
Seroma risk forest plot [19,20,21,22,23,24,25,26,27,28,29,30,31,32,33].

Fourteen studies analyzed the risk of seroma formation, revealing a significant difference between the two groups, with LogOR = −0.90 (95% CI: −1.26 to −0.54). A *p*-value < 0.01 indicates a strong association between the use of electrocautery and an increased risk of seroma formation. The results suggest an average 59% reduction in seroma risk when using the cold cutting technique. As shown in the forest plot, heterogeneity is moderate with an I-squared = 0.35 (Figure 4). Subsequent subgroup analysis of RCT-only studies improved the results in favor of cold cutting (scalpel) with LogOR = −0.95 (95% CI: −1.26 to −0.63), *p*-value < 0.01, and low heterogeneity I-squared = 0.00.

#### 3.3.2. Infection Risk

**Figure 5:**
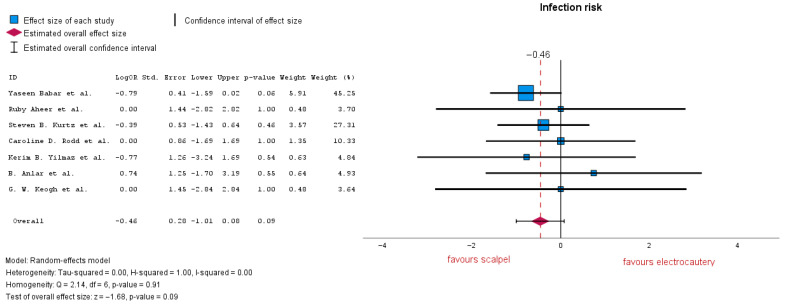
Infection risk forest plot [19,20,22,24,25,30,33].

Seven studies evaluated the risk of infection, demonstrating no significant difference between the two groups, with LogOR = −0.46 (95% CI: −1.01 to 0.08), *p* = 0.09. According to the forest plot, heterogeneity is notably low with an I-squared = 0.00 (Figure 5). The subgroup analysis conducted here on four out of eight studies which reported a 50 mL drain removal decision resulted in LogOR = −0.26 (95% CI: −1.11 to 0.58), *p* = 0.54; analyzing three out of eight studies which reported a 30 mL decision for drain removal resulted in LogOR = 0 (95% CI: −1.35 to −1.35), *p* = 1. Both subgroup heterogeneity analyses resulted in I-squared = 0.00.

#### 3.3.3. Total Volume Drained

**Figure 6:**
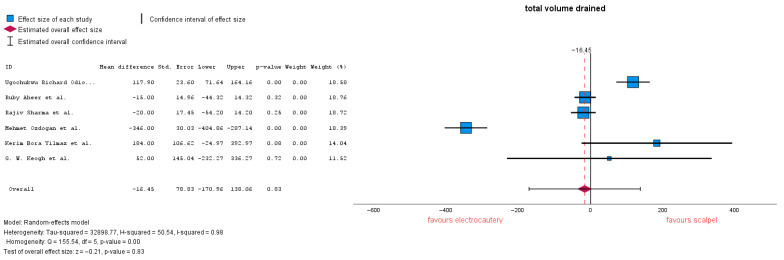
Total volume drained forest plot [20,23,24,28,30,32].

Total volume drained is defined as the amount of fluid removed from the surgical wound after the mastectomy procedure. Six studies analyzed the volume drained after surgery. Of these, Refs. [20,24] reported a decision for drain removal of <50 mL/24 h. No significant differences between the two groups were highlighted, with a mean difference (MD) = −16.45 (95% CI: −170.96 to 138.06), *p* = 0.83. Heterogeneity in this forest plot is markedly high with an I-squared = 0.98 (Figure 6). Subgroup analysis conducted on only studies which reported 30 mL resulted in MD = −64.3 (95% CI: −205.2 to 76.6), *p* = 0.37, and heterogeneity remained high with I-squared = 0.98.

#### 3.3.4. Operation Time

**Figure 7:**
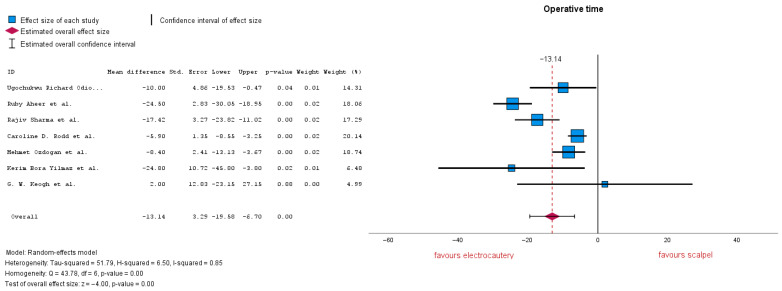
Operation time forest plot [20,22,23,24,28,30,32].

Seven studies evaluated operation time, with an MD = −13.14 (95% CI: −19.58 to −6.70), *p* = 0.00. A *p*-value under 0.01 shows that the result is highly significant. Heterogeneity is markedly high with an I-squared = 0.85 (Figure 7). Subgroup analysis was conducted, removing studies that reported no electrocautery or which did not report other additional coagulation methods, MD = −7.18 (95% CI: −10.85 to −3.51), *p* = 0.00. Heterogeneity lowered to I-squared = 0.27, now being low–moderate.

#### 3.3.5. Blood Loss

**Figure 8:**
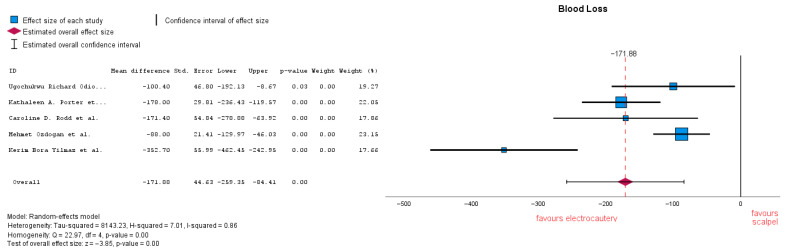
Blood loss forest plot [21,22,23,24,33].

Blood loss estimation varied in several studies through gauze weight or suction volume. Five studies analyzed blood loss, showing that there is a significant difference between the two groups, with an MD = −171.60 (95% CI: −259.35 to −84.41), *p* = 0.00. Heterogeneity in this forest plot is quite high with an I-squared = 0.86 (Figure 8). Subgroup analysis was conducted, removing studies which reported no electrocautery as an additional coagulation method and resulted in MD = −198.55 (95% CI: −348.52 to −48.59), *p* = 0.009. Heterogeneity remained unchanged: I-squared = 0.86.

#### 3.3.6. Duration of Drainage

**Figure 9:**
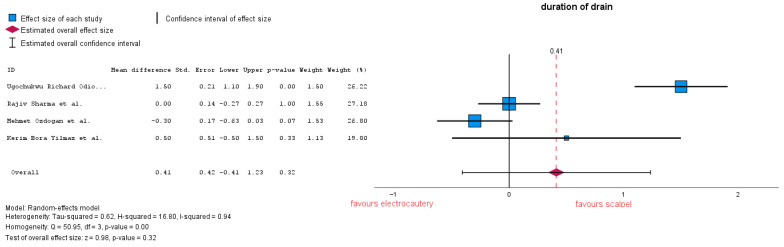
Duration of drainage forest plot [23,24,28,32].

The duration of drainage is calculated as the interval between installation and when the fluid volume collected is below the drain removal decision. Four studies analyzed the duration of drainage, showing a similarity between the two groups, with an MD = 0.4 (95% CI: −0.41 to 1.23), *p* = 0.32. Of the four studies, Ref. [24] reported a decision of drain removal of <50 mL/24 h. Heterogeneity in this forest plot is considerably high with an I-squared = 0.94 (Figure 9). Subgroup analysis conducted on only studies which reported 30 mL resulted in MD = 0.39 (95% CI: −0.56 to 1.34), *p* = 0.42, and heterogeneity remained high: I-squared = 0.96.

## 4. Discussion

### 4.1. Interpretation of Main Findings in Context

When deciding on the usage of “traditional” instruments, surgeons can expect the following results: a lower seroma risk, no difference in total volume drained, or drainage duration. However, this comes at the cost of longer operative time and higher intraoperative blood loss. The analysis of the risk of infection, although not statistically significant, exposed a trend towards lower infection rates when opting for the “cold” technique, potentially due to less thermal injury [40].

Our results align with the previous findings in the literature. For instance, on the subject of seroma risk, the meta-analysis by Oyewale et al. [41] similarly found that cold cutting techniques were associated with lower seroma rates without increasing other complications like total drain amount, reaffirming the position of the safer choice in this regard.

Turner et al. [42] further confirm these findings, underlining the reduction in seroma formation but acknowledging the trade-off in operative efficiency due to longer procedure times.

### 4.2. Heterogeneity and Measurement Challenges

Difficulties in interpreting the results lie in the heterogeneity of the included studies, particularly within demographic outcome groups. Some can be attributed to different methods of measurement; for blood loss, direct measurement is practically impossible, leading to the use of varied indirect methods, such as swab weight or suction volume, introducing variability.

Odionyeme et al.’s [23] was the only study that did not use electrocautery as an additional method of coagulation and reported lower blood loss. Subgroup analysis was performed, limited to studies employing electrocautery for coagulation, but no other significant results could be derived.

Similarly, Keogh [20] was an outlier in operative time, so the authors assume the presence of a differing surgical protocol; nonetheless, in the Methods Section of the study, it is clearly stated that the procedure was pectoralis-sparing. While this introduced variation, the trend of longer operating times with traditional tools remained consistent.

### 4.3. Limitations

Despite meaningful findings, several limitations must be acknowledged.

Only one study [33] reported standardized PROMs (Patient-Reported Outcome Measures), limiting insight into patient-centered outcomes such as pain, satisfaction, and cosmesis.

Secondly, the lack of significant findings across the hematoma, flap necrosis, and ecchymosis must be interpreted with caution. The small sample sizes and the limited number of studies reporting these complications likely contributed to the wide confidence intervals and reduced statistical power.

High heterogeneity was encountered in the drainage output and drainage duration outcomes. After subgroup analysis based on drainage removal criteria, it remained similar and did not affect the conclusion. Such results are also found in the literature [43].

Two studies [23,24] also reported cytokine levels in drainage fluid, as a biomarker of tissue trauma and inflammatory response. No statistical analysis could be conducted due to incomplete data reporting; one study presented only graphical data, although, presumptively, both suggested that modified radical mastectomy using the “cold” technique results in a less aggressive tissue response. This topic must be further researched to confirm this hypothesis.

### 4.4. Future Research Directions

Future studies should prioritize standardized definitions and outcome measures, particularly in operative time and blood loss estimation. Additionally, routine inclusion of PROMs, including validated scales such as the VAS (Visual Analogue Scale) for pain, would enhance the relevance of findings for both clinicians and patients. While clinical endpoints are important, future studies should incorporate patient-reported pain, satisfaction, and cosmetic outcomes to inform shared surgical decision-making.

Moreover, the preliminary evidence on cytokine modulation through surgical technique represents an intriguing avenue that warrants deeper biochemical exploration with larger cohorts and standardized assays.

## 5. Conclusions

Traditional (“cold”) surgical techniques in mastectomy are associated with reduced seroma formation and potentially lower infection rates. However, these benefits come at the cost of longer operative times and higher intraoperative blood loss compared to electrocautery. Thus, electrocautery should be employed in patients who benefit from the shorter operative time and lower blood loss, while patients in better clinical condition should benefit from cold cutting techniques. Further research that focuses on patient-reported outcomes and deeper investigation into inflammatory biomarkers (e.g., cytokines) may help shift the balance in surgical decision-making.

## Figures and Tables

**Table 1:** Study characteristics [19,20,21,22,23,24,25,26,27,28,29,30,31,32,33].

Authors	Year	Design	LoE	Use of Additional Method of Coagulation in Scalpel Group	Number of Patients			AGE (yr)		BMI		Level of Lymph Node Harvest	Drain Removal Decision
					Total	CC	EC	C	EC	C	EC		
Babar et al. [33]	2024	RCT	1b	N/R	240	120	120	53.1 ± 8.2	52.3 ± 7.5	27.1 ± 3.9	26.4 ± 4.2	N/R	N/R
Odionyeme et al. [32]	2024	RCT	1b	No electrocoagulation	46	23	23	45 ± 12.8 (25–73)	49.1 ± 11.4 (27–66)	25.6 ± 3.5	24.8 ± 2.6	I/II	<30 mL/24 h
Bashir et al. [31]	2023	RCT	1b	N/R	100	50	50	42.39 ± 8.3 (22–60)		N/R	N/R	N/R	N/R
Aheer et al. [30]	2023	RCT	1b	N/R	60	30	30	N/R	N/R	N/R	N/R	N/R	<30 mL/24 h
Zim et al. [29]	2019	prospective cohort	2b	Electrocoagulation	90	35	55	52	53	(18.5–30)	(18.5–30)	I/II/III	<30 mL/24 h
Sharma et al. [28]	2018	RCT	1b	N/R	70	35	35	N/R	N/R	N/R	N/R	N/R	<30 mL/24 h
Chavan et al. [27]	2017	RCT	1b	Electrocautery	176	67	109	53 (40–65)	52(40–65)	(18.5–30)	(18.5–30)	I/II/III	<30 mL/24 h
Ali et al. [26]	2014	RCT	1b	N/R	40	20	20	N/R	N/R	N/R	N/R	N/R	<30 mL/24 h
Anlar et al. [25]	2013	RCT	1b	Harmonic scalpel	81	40	41	51 (29–68)	52 (24–82)	N/R	N/R	I/II	<50 mL/24 h
Yilmaz et al. [24]	2011	RCT	1b	Electrocoagulation	53	27	26	50.2 ± 9.3	48.5 ± 9.8	27.9 ± 0.88	28.1 ± 0.75	II/III	<50 mL/24 h
Özdogan et al. [23]	2008	prospective cohort	2b	Electrocoagulation	38	20	18	50.1 ± 2.32	50.2 ± 3.43	27.4 ± 0.98	26.11 ± 0.86	III	<30 mL/24 h
Rodd et al. [22]	2007	RCT	1b	Electrocoagulation	60	30	30	67.03 ± 8.69	69.6 ± 9.8	N/R	N/R	I	<30 mL/24 h
Porter et al. [21]	1998	RCT	1b	Electrocoagulation	80	38	42	63 ± 13	66 ± 14	26 ± 5	28 ± 9	N/R	<30 mL/24 h
Keogh et al. [20]	1998	RCT	1b	Electrocoagulation	42	21	21	60 (45–78)	60 (41–85)	N/R	N/R	III	<50 mL/24 h
Kurtz et al. [19]	1995	retrospective cohort	2b	N/R	196	86	110	56.5 (30–80)	55.9 (29–89)	N/R	N/R	III	<50 mL/24 h

Data presented as mean ± standard deviation (min–max); Abbreviations: LoE, Level of Evidence; NR, not reported; RCT, randomized controlled trial; CC, cold cutting; EC, electrocoagulation.

**Table 2:** Demographical outcomes of included studies.

Demographics	Electrocoagulation	Cold Cutting	Mean Difference	(*p*) Value
Age [19,20,21,22,25,27,29,30,31,38,39]	55.7 ± 9.52	54.9 ± 9.42	0.07	0.48
BMI [19,21,22,30,31]	26.7 ± 4.99	26.9 ± 3.69	−0.47	0.55
Stage of disease 0/I/II/III [19,20,22,25,27,30,39]	18%/35%/27%/28%	13%/31%/33%/30%	N/A	N/A
Number of collected lymph nodes [19,21,38]	13.6 ± 7.01	14.4 ± 6.28	−0.62	0.23
Number of positive lymph nodes [19,21]	2.5 ± 2.85	1.1 ± 2.45	2.27	0.31
follow-up period [25,26,27,28,30,31]	53.8	48.1	N/A	N/A
% of hypertensive patients [22,30,31]	30%	26%	N/A	N/A
% of smokers [22,31]	18%	16%	N/A	N/A
% of diabetes [22,31]	20%	20%	N/A	N/A

Data presented as pooled weighted mean ± pooled standard deviation. Abbreviations: N/A not applicable

**Table 3:** Secondary outcomes of included studies.

Outcome	Electrocautery	Cold Cutting	Mean Difference	(*p*) Value
specimen weight (g) [20,23,38]	604.5 ± 372.63	657.6 ± 425.193	−47.26	0.49
specimen volume (mL) [21,22,23]	2324.1 ± 401.58	2083.7 ± 531.84	316.74	0.15
seroma volume (mL) [21,31]	68.9 ± 18.89	51.5 ± 22.25	24.47	0.054
flap area (mm^2^) [22,23]	493.7	460.8	N/A	N/A
hospital stay (days) [31,38]	4.3	4.2	N/A	N/A

Data presented as pooled weighted mean ± pooled standard deviation (min–max). Abbreviations: N/A not applicable

**Table 4:** Secondary complications of included studies.

Complication	OR (Odds Ratio)	95% CI	(*p*) Value
Hematoma [20,22,23,30,39]	1.17	0.38–3.5	0.77
Flap necrosis [22,23,26,28]	1.56	0.67–3.62	0.29
Ecchymosis [22,23]	0.7	0.34–1.47	0.35

## Data Availability

The raw data supporting the conclusions of this article will be made available by the authors on request.
